# Supplementary material for: An Assessment of a New Rapid Multiplex PCR Assay for the Diagnosis of Meningoencephalitis
Source: Diagnostics (Basel). 2024 Apr 11;14(8):802. doi: 10.3390/diagnostics14080802 (PMC11048994; doi:10.3390/diagnostics14080802)
Supplement: Supplementary file 1 [file diagnostics-14-00802-s001.zip › diagnostics-2920751-supplementary.pdf]

Supplementary Materials

- Detection of remnants of past VZV ME

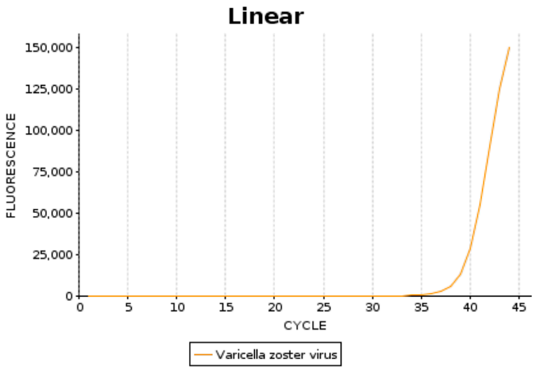

Detected ⊕ **Varicella zoster virus**

**User**	administrator	**Test Status**	Completed
		**Internal Controls**	Passed

RESULT DETAILS			Ct / EP
**Viruses**	Not detected	Enterovirus	– / –
	Not detected	Herpes simplex virus 1	– / –
	Not detected	Herpes simplex virus 2	– / –
	Not detected	Human parechovirus	– / –
	Not detected	Human herpes virus 6	– / –
	Detected	Varicella zoster virus	38.5 / 150,569
**Bacteria**	Not detected	*Streptococcus pneumoniae*	– / –
	Not detected	*Neisseria meningitidis*	– / –
	Not detected	*Streptococcus agalactiae*	– / –
	Not detected	*Listeria monocytogenes*	– / –
	Not detected	*Haemophilus influenzae*	– / –
	Not detected	*Escherichia coli K1*	– / –
	Not detected	*Streptococcus pyogenes*	– / –
	Not detected	*Mycoplasma pneumoniae*	– / –
**Fungi & Yeast**	Not detected	*Cryptococcus neoformans/gattii*	– / –
**Controls**	Detected	IC	33.0 / 294,354

- Active VZV encephalitis

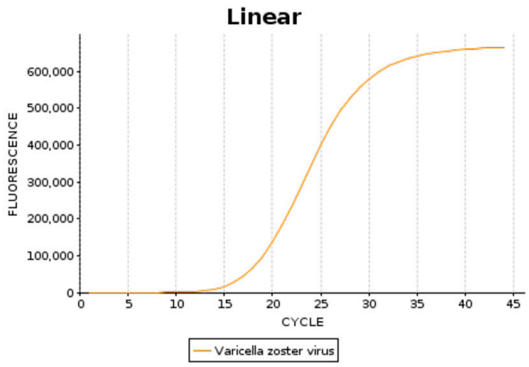

Detected ⊕ **Varicella zoster virus**

**User**	administrator	**Test Status**	Completed
		**Internal Controls**	Passed

RESULT DETAILS			Ct / EP
**Viruses**	Not detected	Enterovirus	– / –
	Not detected	Herpes simplex virus 1	– / –
	Not detected	Herpes simplex virus 2	– / –
	Not detected	Human parechovirus	– / –
	Not detected	Human herpes virus 6	– / –
	Detected	Varicella zoster virus	15.4 / 665,144
**Bacteria**	Not detected	*Streptococcus pneumoniae*	– / –
	Not detected	*Neisseria meningitidis*	– / –
	Not detected	*Streptococcus agalactiae*	– / –
	Not detected	*Listeria monocytogenes*	– / –
	Not detected	*Haemophilus influenzae*	– / –
	Not detected	*Escherichia coli K1*	– / –
	Not detected	*Streptococcus pyogenes*	– / –
	Not detected	*Mycoplasma pneumoniae*	– / –
**Fungi & Yeast**	Not detected	*Cryptococcus neoformans/gattii*	– / –
**Controls**	Detected	IC	33.8 / 219,794

Figure S1. Difference between the case to be interpreted as the detection of remnants of past VZV ME Ct and that of active VZV encephalitis.
